# The Treatment of Incipient Madness

**Published:** 1918-10-19

**Authors:** George M. Robertson

**Affiliations:** Royal Asylum, Morningside, Edinburgh.


### THE EDITOR'S LETTER-BOX.

**[Correspondence on all subjects is invited, but we cannot in any way be responsible for the opinions expressed by our correspondents, who must give their name and address as a guarantee of good faith, but not necessarily for publication. Correspondents are reminded that brevity of style and conciseness of statement greatly facilitate early insertion.]**

### The Treatment of Incipient Madness.

*To the Editor of* THE HOSPITAL.

SIR,—There is a statement in the leading article of THE HOSPITAL on the "Treatment of Incipient Madness" in the number published on October 5 which, so far as Scotland is concerned, is not strictly accurate.

The writer of the article, referring to the admission of voluntary patients into lunatic asylums, states that at present this beneficial provision is granted to private patients but is denied to rate-supported patients. There is no legal obstacle to the admission of rate-supported patients as voluntary patients or boarders into the Scottish asylums. I have at present one such patient under my care.

The difficulty with regard to the admission of these patients is due to another reason altogether—namely, the desire to keep down the rates and the objection to incur liability for the maintenance of any patient requiring care in an asylum unless, being legally certified to be insane, his maintenance is compulsory under the Lunacy Acts. It is within the power of the Inspector of Poor and the Parish Council to incur expense for the provision of such medical care and treatment as any patient requires. If it be necessary, for example, to call in a surgeon to perform a special operation this can be done. Similarly, if it be necessary for the proper care and treatment of a patient that he should enter an asylum as a voluntary patient, the expenditure for this purpose can also be incurred. I am, etc.,

GEORGE M. ROBERTSON, M.D., F.R.C.P. Edin.

Royal Asylum, Morningside, Edinburgh.
October 11, 1918.

